# Patterns of dementia treatment in older adults with Parkinson’s disease using nationwide medical claims data

**DOI:** 10.1186/s12877-022-03028-0

**Published:** 2022-04-22

**Authors:** Bora Yoon, Hwa-Jung Kim

**Affiliations:** 1 Department of Neurology, Konyang University College of Medicine, Konyang University Hospital, Daejeon, South Korea; 2 Department of Preventive Medicine, Ulsan University College of Medicine, Seoul, South Korea; 3 Department of Clinical Epidemiology and Biostatistics, ASAN Medical Center, Ulsan University College of Medicine, 88 Olympic-ro 43-gil, Songpa-gu, Seoul, 05505 South Korea

**Keywords:** Parkinson’s disease, Dementia, Korea, Claims data

## Abstract

**Background:**

Dementia is a common feature in Parkinson’s disease (PD); however, data on dementia treatment patterns in patients with PD are scarce. This study aimed to evaluate the incidence of dementia in individuals with PD and to describe the dementia treatment patterns in the Korean elderly population.

**Methods:**

We conducted a retrospective population-based cohort study using data obtained from the Korean National Health Insurance Service-Senior Cohort (NHIS-SC) database. The dataset comprised more than 500,000 health insurance beneficiaries from January 1, 2002 to December 31, 2015. We estimated the incidence of patients newly diagnosed with dementia during this observational period, compared patient demographics, and analyzed the exposure to anticholinergic drugs among PD patients with (PD + D) and without (PD-D) dementia. Furthermore, the duration to dementia diagnosis and patterns of dementia treatment were evaluated.

**Results:**

A cohort of 28,537 patients aged 60 years or older who were diagnosed with PD by the NHIS was established. Within this cohort, 8620 patients were eligible study participants according to strict inclusion/exclusion criteria. Of these individuals, 3879 (45.0%) patients were newly diagnosed with dementia; the incidence of dementia in PD was 15.2 per 1000 person-years. The proportion of women was higher in the PD + D (64.6%) than the PD-D group (58.2%) (*P* < 0.001); furthermore, the use of anticholinergic medication was greater in PD + D (37.6%) than in PD-D (24.0%) patients. The incidence curves for dementia over time were the steepest during the first year and decreased every year thereafter. Approximately 60% of PD patients were diagnosed with dementia during the first 3 years. Regarding the use of anti-dementia drugs, 2539 (65.5%) of 3879 PD + D were prescribed medication. During the observation period, 1799 (70.9%) patients were prescribed only one type of anti-dementia drug. In this monotherapy group, the most commonly prescribed medication was donepezil (1313[73.0%]), followed by rivastigmine (capsule and patch; 246[13.7%]), memantine (187[10.4%]), and galantamine (53[2.9%]).

**Conclusions:**

In Korea, dementia was observed to occur relatively soon after the diagnosis of PD. Anti-dementia medication was prescribed to approximately 66% of PD + D patients, with the majority receiving donepezil as monotherapy.

**Supplementary Information:**

The online version contains supplementary material available at 10.1186/s12877-022-03028-0.

## Introduction

Parkinson’s disease (PD) is a common, complex, and slowly progressive neurodegenerative disease characterized by classical parkinsonian motor symptoms, such as tremor, rigidity, bradykinesia, and postural instability [1]. In 2016, an estimated 6.1 million individuals globally were diagnosed with PD, which was 2.4 times higher than in 1990 [2]. The incidence of PD increases with age and is uncommon among individuals younger than 50 years of age [2]. According to the Korean National Health Insurance Service (NHIS) claims data from 2012 to 2015, the incidence of PD increased from 156.9 per 100,000 persons in 2012 to 181.3 per 100,000 persons in 2015 [3].

Non-motor symptoms, including olfactory, autonomic and cognitive dysfunction, pain, and psychiatric and sleep disturbances are also characteristic of PD [4, 5]. Some non-motor symptoms may even occur during the prodromal stages of the disease, worsen with disease progression, and surpass motor symptoms as the major factors that affect the patient’s quality of life and caregiver burden. In particular, cognitive and behavioral symptoms are highly prevalent in PD; additionally, their cumulative incidence increases with disease progression. The incidence of dementia in PD patients was reported to be 30–40%, and the incidence rate is five to six times that in age-matched healthy controls [6]. Furthermore, the cumulative incidence of dementia over 8 years was reported to be as high as 78% [7]; the incidence of dementia 12 years after PD diagnosis is observed to be between 80 and 90% [8]. Despite the high incidence of dementia in PD, the evidence for the medical treatment of dementia in patients with PD is less established than that for Alzheimer’s disease (AD).

Systematic reviews reported limited evidence wherein acetylcholinesterase inhibitors (AChEIs) and memantine slightly improved the global impression; however, only AChEIs enhanced the cognitive function [9–11]. Among the AChEIs, only rivastigmine is considered to be “efficacious and clinically useful”, whereas donepezil and galantamine are considered to be “possibly useful” because of insufficient evidence [12]. For memantine, practice implications have been investigational with insufficient evidence. However, in addition to evidence, approval indication from the Korea Ministry of Food and Drug Safety, the ease of use, or adverse events would have influenced the actual treatment by clinicians in the real world. The rivastigmine patch, for example, has a broader treatment spectrum with approval to treat mild to severe dementia, whereas rivastigmine capsules have been approved to treat only mild to moderate dementia. Donepezil is taken once a day; however, rivastigmine is taken twice a day. Patches, in general, require far more care and assistance from caregivers than oral medications. It is well known that the adverse event rates increase proportionately with drug dosage. In AD, 6–12 mg/day dosage of rivastigmine was superior to placebo, while 1–4 mg/day was not [13], which suggests that there may be a greater risk of adverse events to reach an effective dose. These factors, together, must be taken into account when considering the treatment of PD with dementia (PD + D) patients in real-world practice. The goal of the present study was to investigate the incidence of dementia in patients with PD and to describe the dementia treatment patterns in Korea.

## Methods

All methods were performed in accordance with the Declaration of Helsinki.

### Data source

The data used in this study were extracted from the National Health Insurance Service-Senior Cohort (NHIS-SC) database (version 3.0). The NHIS is the compulsory universal health insurance system in South Korea that covers about 52 million (97%) people in the Korean population. The NHIS-SC is a retrospective data cohort comprising 14 years (January 1 2002– December 31 2015) of cumulative records of 558,147 health insurance beneficiaries. These individuals (5.5 million) were randomly selected with a sampling ratio of 10% from the senior population, who were aged 60 years or older in 2002. The profile was described in detail [14]. The details about the database can be obtained from the NHI Sharing Service (https://nhiss.nhis.or.kr/bd/ab/bdaba015lv.do).

The NHIS-SC database contains information on personal demographics, socioeconomic status, diagnoses as coded by the tenth edition of the International Classification of Diseases, 10th version (ICD-10), procedures, and drug prescriptions.

### Study design and population selection

**Fig. 1:**
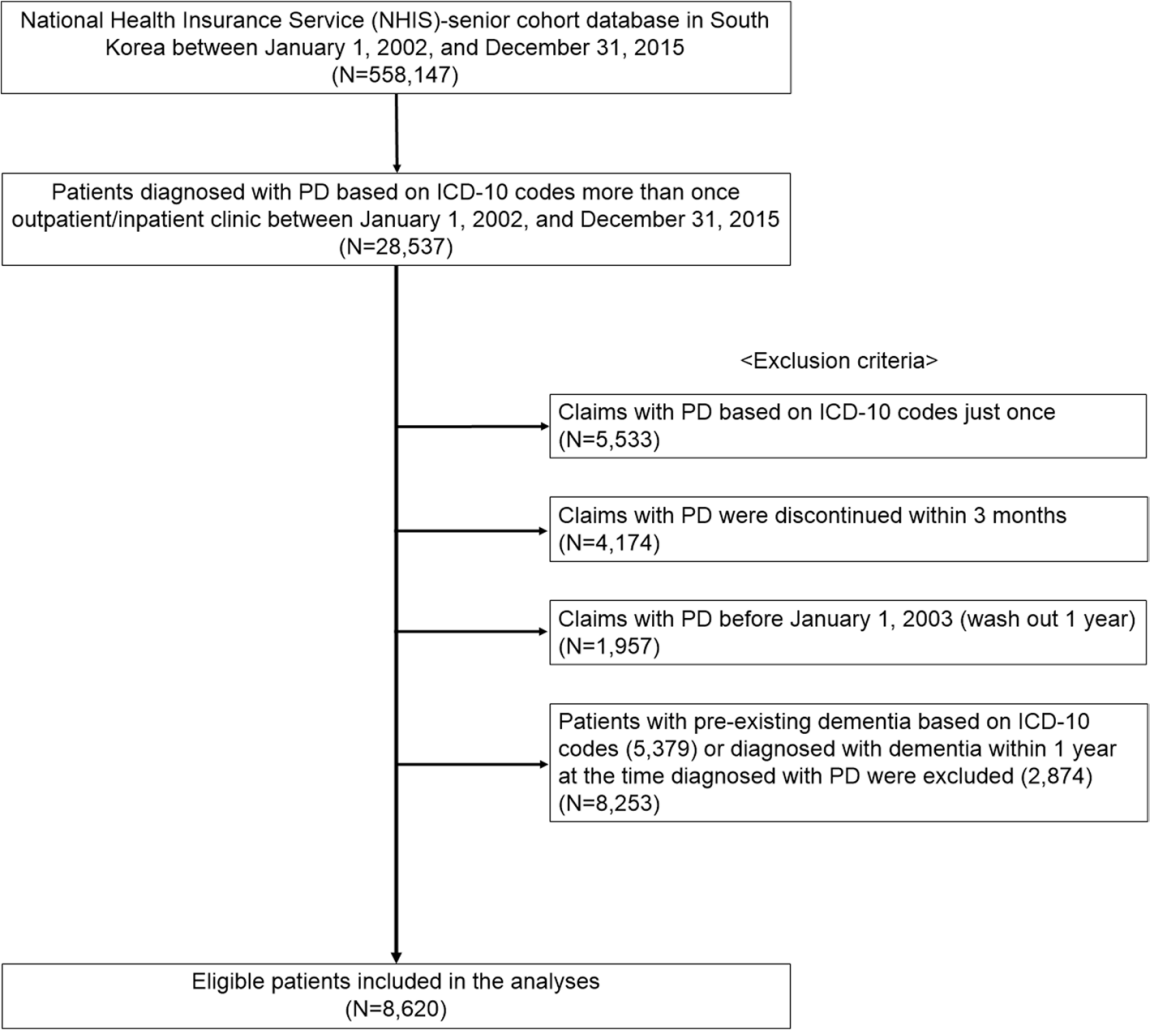
Flow chart of the study population. Abbreviations: PD, Parkinson’s disease; ICD-10, International Classification of Diseases, 10th revision

A retrospective cohort of patients who were diagnosed with PD in the NHIS-SC was established. This diagnosis was based on the ICD-10 code for PD (G20) and dementia (F00, F01, F02, F03, and G30). Given that the primary aim of this study was to estimate the incidence of dementia among PD patients, we used strict working definitions to eliminate patient misclassification: PD patients with fewer than two claims and patients who discontinued anti-parkinsonian medication within 90 days were excluded. To reduce the possibility of secondary parkinsonism, the patients with first claims for PD within 1 year of the cohort index date (the inclusion date into the NHIS-SC) considering the washout period were excluded. The association between PD and dementia was estimated after exclusion of PD patients with pre-existing dementia or patients with claims for dementia within 1 year of claims for PD to minimize the inclusion of the patients with dementia with Lewy bodies, progressive supranuclear palsy, corticobasal syndrome, vascular parkinsonism, drug-induced parkinsonism, CO poisoning, normal pressure hydrocephalus, or frontotemporal dementia with parkinsonism. The flowchart showing the patient selection is presented in Fig. 1.

### Outcome and confounder variables

The primary objective was to evaluate the incident dementia cases among PD patients who with new claims for any health insurance with dementia diagnosis (Table 40 of NHIS-SC), which is defined as any health insurance claim with a diagnosis of dementia after at least 1 year of being diagnosed with PD. The incidence of dementia among individuals with PD was calculated during the follow-up period as follows: (1) from the date of the first health insurance claim with PD, as the index date, and (2) until the earliest date regarding the date of the first health insurance claim with dementia, date of death, or end of the study observation period (December 312,015); the observation period would be a maximum of 13 years. We also estimated the duration from the diagnosis of PD to the diagnosis of dementia. The prescription of any dementia medication (prescription or not), pattern of the anti-dementia drug (i.e., donepezil, rivastigmine, galantamine, and memantine), and exposure to anticholinergic agents (i.e., trihexyphenidyl and benztropine) were examined in PD + D patients. We also identified the first prescription date for the anti-dementia medication and calculated the duration between the first health insurance claims of PD diagnosis and the first prescription date of anti-dementia medication. Sociodemographic data, such as age at the first health insurance claim with PD diagnosis, gender, residential area, socioeconomic status, and the use of anticholinergic medications were evaluated.

### Statistical analysis

Descriptive statistics were calculated: the overall dementia incidence rate among PD was estimated per 100,000 person-years; additionally, the demographics of PD without dementia (PD-D) and PD + D were compared using a *t*-test or chi-square test. Additionally, standardized differences [15] were calculated to determine the balance of baseline covariates between the two groups. All statistical analyses were performed using SAS version 9.4 (SAS Institute, Cary, USA); a *P*-value < 0.05 was considered to be significant.

## Results

### Population characteristics

The characteristics of all 8620 participants are displayed in Table 1. The PD + D group (64.6%) had a higher proportion of women than the PD-D group (58.2%) (*P* < 0.001). The mean age at the first health insurance claim with PD diagnosis was similar in both groups (74.6 vs. 74.0); however, the proportion of individuals in their 60s was higher in the PD + D group. Furthermore, the proportion of people living in rural areas and receiving medical aid was higher in the PD + D group. The number of patients prescribed anticholinergic medication was higher in the PD + D group (PD-D, 24.0% vs. PD + D, 37.6%; *P* < 0.001).

**Table 1:** Characteristics of the 8620 study participants

	All (*n* = 8620)			
Characteristic	PD without D (*n* = 4741)	PD with D (*n* = 3879)	*P*	*SMD*
Sex, female	2760 (58.2)	2505 (64.6)	< 0.001	0.1316
Age at the first claim with PD diagnosis (years)	74.6 ± 5.9	74 ± 6.0	< 0.001	0.1009
60–64	145 (3.1)	174 (4.5)		
65–69	781 (16.5)	756 (19.5)		
70–74	1556 (32.8)	1216 (31.4)		
75–79	1319 (27.8)	1033 (26.6)		
80–84	660 (13.9)	517 (13.3)		
85 or more	280 (5.9)	183 (4.7)		
Residential area			< 0.001	
Seoul and capital area^a^	2003 (42.3)	1401 (36.1)		0.1271
Metropolitan city^b^	830 (17.5)	700 (18.1)		0.0157
Rural area	1908 (40.2)	1778 (45.8)		0.1132
Income quintiles			< 0.001	
Medicaid	549 (11.6)	597 (15.4)		0.1115
Q1(lowest)	589 (12.4)	458 (11.8)		
Q2	421 (8.9)	336 (8.7)		
Q3	521 (11)	416 (10.7)		
Q4	849 (17.9)	628 (16.2)		
Q5(highest)	1812 (38.2)	1444 (37.2)		
Anticholinergic use	1138 (24.0)	1459 (37.6)	< 0.001	0.2962
Trihexyphenyl	711 (15.0)	910 (23.5)	< 0.001	0.2168
Benztropine	524 (11.1)	778 (20.1)	< 0.001	0.2505
Anti-dementia medication use	15 (0.3)	2539 (65.5)	< 0.001	1.7764

*Abbreviations*: *PD* Parkinson’s disease, *D* dementia, *SMD* standardized mean difference

Data are shown as mean ± SD or number (%). *P* values were obtained by *t*-test for continuous variables or chi-square test for categorical variables

^a^Seoul, Incheon, and Gyeonggi-do; ^b^Busan, Daegu, Daejeon, Gwangju, Sejong, and Ulsan

### Incidence of dementia in PD

**Fig. 2:**
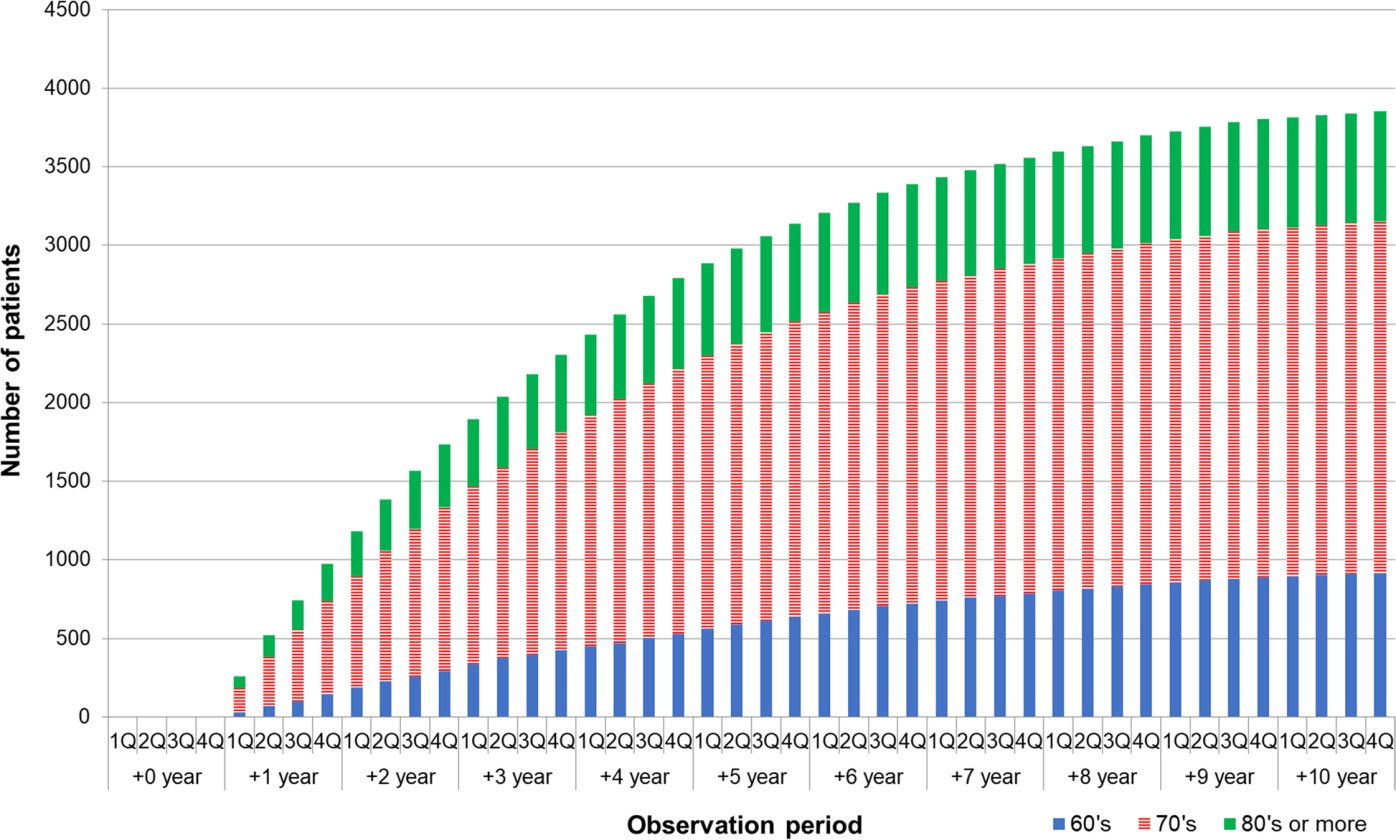
Incidence of dementia in Parkinson’s disease (PD) over time. The X-axis represents the quarter (Q) and year, and the Y-axis represents the number of people. The slope of the first year was the steepest, and decreased every year thereafter. Approximately 25% of patients were diagnosed with dementia within the first year; approximately 60% of patients were diagnosed within the first 3 years, and more than 80% of patients were diagnosed within the first 5 years. The higher the age group, the more rapid the diagnosis of dementia

The median follow-up duration (inter-quartile range, IQR) was 6.2 (3.0 ~ 9.3) and 3.3 (2.0 ~ 5.3) years in the PD and PD + D groups, respectively. The entire observational period comprised 44,828 person-years (median [IQR] = 4.5[2.3–7.7]). During this period, 3879 (45.1%) of the 8620 patients had newly developed dementia, as defined by the F00, F01, F02, F03, and G30 diagnosis codes in PD patients. In our study, the incidence of dementia was 86.5 per 1000 person-years in the study population. The cumulative numbers of dementia in PD patients increased over time. Given the trend in the incidence of dementia (Fig. 2), when comparing the incidence slope annually, the first year is the steepest, and the slope decreases every year thereafter. Approximately 25% of the patients were diagnosed with dementia within the first year; approximately 60% of the patients were diagnosed within the first 3 years, and more than 80% of patients were diagnosed within the first 5 years (Supplementary Table 1). The incidence rate of dementia was different depending on the age group (Fig. 2). With respect to the age group, in their 60s, the incidence of dementia was 15.7% within the first year, 31.6% within the second year, and 46.0% within the third year, which means the incidence rate of dementia for 3 years after PD diagnosis relatively linearly increased and decreased steadily thereafter (Supplementary Table 1). In patients in their 70s, the incidence rate of dementia was high in the first 2 years (26.4% of PD patients within the first year, 46.2% within the second year) and gradually decreased thereafter (Supplementary Table 1). In patients in their 80s and above, 33.6% of PD patients had already been diagnosed with dementia in the first year, showing the highest diagnosis rate of dementia in the first year (Supplementary Table 1).

### Use of anti-dementia medication in PD

In PD + D patients, 2539 (65.5%) of 3879 individuals were prescribed anti-dementia drugs, while 1340 (34.5%) patients were not. The prescription patterns of anti-dementia medication are shown in Table 2. During the entire observational period, 1799 (70.9%) of 2539 individuals were prescribed only one anti-dementia medication (or monotherapy). The most commonly prescribed drug was donepezil (1951 patients, 65.2%), followed by rivastigmine (capsule and patch combined: 753 patients, 25.1%), memantine (563 patients, 18.8%), and galantamine (186 patients, 6.2%). The first medication prescribed was donepezil, followed by memantine, rivastigmine patch, rivastigmine capsule, and galantamine. When the number of patients who were first prescribed drugs was compared with that of patients on monotherapy throughout the entire observation period, donepezil (78.2%) had the highest prescription persistence rate; this was followed by memantine (61.1%), rivastigmine patch (57.5%), rivastigmine capsule (51.9%), and galantamine (40.5%). Of 2539 patients, 740 (29.1%) patients were prescribed more than two anti-dementia drugs during the observational period; however, only 26 patients (1.0%) started more than two drugs as the first drug chosen, implying that most patients who were prescribed more than two anti-dementia drugs were switched from one to a different anti-dementia drug, or combined later.

**Table 2:** Prescribing pattern of anti-dementia medications

	Number (%)	Number (%)	Number (%)
Number of drugs for the entire observational period	Total	Single	Multiple
	2539 (100)	1799 (70.9)	740 (29.2)
The proportion of each drug among			
Donepezil	1951 (76.9)	1313 (73.0)	638 (86.3)
Rivastigmine patch	423 (16.7)	138 (7.7)	285 (38.6)
Rivastigmine capsule	330 (13.0)	108 (6.1)	222 (30.0)
Galantamine	186 (7.4)	53 (3.0)	133 (18.0)
Memantine	563 (22.2)	187 (10.4)	376 (50.9)
First prescribed drug			
Donepezil	1680 (66.2)	1313 (73.0)	367 (49.6)
Rivastigmine patch	240 (9.5)	138 (7.7)	102 (13.8)
Rivastigmine capsule	208 (8.2)	108 (6.1)	100 (13.6)
Galantamine	131 (5.2)	53 (3.0)	78 (10.6)
Memantine	306 (12.1)	187 (10.4)	119 (16.1)

### Use of anticholinergic medication in PD

Anticholinergic medication was prescribed to 2597 (30.1%) of 8620 patients with PD; trihexyphenidyl was prescribed more than benztropine (Table 1). Anticholinergic use was observed to be higher in PD + D (37.6%) than in PD-D (24.0%) (*P* < 0.001) (Table 1). The number of PD patients concurrently prescribed anticholinergic and anti-dementia medications was 1006 (25.9% of PD + D patients.

## Discussion

The present study sheds light on dementia treatment in PD patients. Specifically, we determined the number of PD patients diagnosed with dementia and the number of patients who were prescribed anti-dementia medication and identified the patterns of anti-dementia drug use. In our study, the incidence of dementia in PD was 15.2 per 1000 person-years. This could reflect the incidence in the Korean elderly over 60 years of age and will be more informative than the incidence of PD across all age groups. This is because PD is uncommon among individuals younger than 50 years; additionally, its incidence increases with age [2].

Approximately 45% of PD patients were diagnosed with dementia, consistent with previous findings that 25–40% of patients with PD eventually develop dementia [16]. The result that approximately 65% of PD + D group were women, which was definitely higher proportion compared to the PD-D group, is similar to the fact that 2/3 were women in AD [17]. This suggests that the general factor that men are more likely to develop PD than women may not apply equally to PDD. Like in AD, a reason for the higher prevalence of women in PD + D may be that they live longer, on average, than men. It is well known that dementia increases over time in PD; however, little research has been done into when dementia is most frequently diagnosed. Our findings revealed a cumulative effect of dementia over time; dementia, however, did not occur linearly over time. In the past, dementia was known at least years after diagnosis of PD, but it is not uncommon for patients with PD to already present with some level of cognitive decline at the time of diagnosis [18]. Our data showed that dementia occurred relatively early in large numbers of cases, approximately 60% within 3 years, and more than 80% within 5 years after PD diagnosis. This suggests that dementia may be associated with PD at a relatively early stage. The data revealed that advanced age in patients with PD was associated with a more rapid diagnosis of dementia. This implies that even in PD, age is an independent risk factor for dementia.

During the observational period, approximately 66% of PD + D patients were treated with the anti-dementia drugs; this was substantially higher in Korea than in the U.S. (27.2%) [19]. However, a simple comparison is not appropriate due to different medical systems and backgrounds. We confirmed that several clinicians in Korea actively considered using anti-dementia medications to treat PD + D patients. Notably, in terms of prescription patterns, we found that donepezil was used to a greater extent than rivastigmine, despite greater clinical evidence with rivastigmine. This result contradicted a previous report that showed that rivastigmine was the most frequently used drug for PD + D patients in Korea [20]; this, however, may be explained by the smaller sample sizes and by the fact that the study participants were enrolled from only 12 university hospitals. This was in concordance with another study that found that donepezil was the most commonly prescribed medication, followed by memantine and rivastigmine [19]. Plausible reasons are that donepezil has a broader spectrum of treatment (mild to severe dementia), can be taken once a day, and has a simpler titration schedule such that reaching a target dose is relatively easy. We found that the proportion of rivastigmine patch use during the entire observational period, or in just one drug prescription, was higher than that for capsule use. We could conjecture that patches have fewer gastrointestinal adverse events and a broader approval spectrum for treating mild to severe dementia; are easier to titrate (i.e., 5, 10, and 15 at least every 4 weeks in patches versus 3, 6, 9, and 12 at least every 2 weeks in capsules, if tolerant); and are simpler to use (once versus twice a day) than rivastigmine capsules. These factors together help to reach a target dose. Memantine has been prescribed for treating PD + D patients, despite the slight lack of evidence that memantine improves cognitive function more than AChEIs in PD + D patients. Memantine has a lower gastrointestinal adverse event profile than AChEIs [21] and a good safety profile for treating dementia in PD [11]. According to the most recent meta-analysis [10], memantine and AChEIs influence the global cognitive function, attention, processing speed, executive functions, memory, and language. In terms of adverse events, only the rivastigmine group experienced more adverse events than the placebo group; donepezil and memantine, however, did not produce any substantial adverse events [10]. Given this evidence, memantine may be an alternative option for treating PD + D patients who experience adverse events with AChEIs.

Co-administration of a AChEI and drug with high anticholinergic activity is a clear prescription error because these drugs produce opposing pharmacologic effects. This is supported by numerous studies on the negative effect of anticholinergic drugs on cognition [22–25]. However, in reality, in PD patients with tremors as a primary symptom, the use of anticholinergic drugs is unavoidable; co-administration of anticholinergic and anti-dementia medication, therefore, is bound to occur. The proportion of concomitant prescriptions of anticholinergic and anti-dementia medications was 25.9% in PD + D, similar to the percentage of anticholinergic medication in PD-D (24.0%). The proportion of anticholinergic medication in PD + D was higher than in PD-D, suggesting that it could be a potential confounding factor for dementia.

There are several limitations to the present study. First, we only evaluated the use of anticholinergic drugs without considering the anticholinergic burden, e.g., how much and for how long high-potency anticholinergic medications have been used, starting time, or duration of use. Studies armed with this information would be better positioned to identify the causal mechanism. Second, we did not collect data on concurrent medications known to cause cognitive dysfunction, such as antipsychotics, sedatives, and antidepressants, nor on potential medications that provoke drug-induced parkinsonism, including typical and atypical antipsychotics or antiemetics. Third, as this study used Korean claims data, there might be limitations in the generalizability to other geographic areas or other races. Forth, due to the completeness of diagnosis among claims data, there might be some underestimation of the incidence. Despite these limitations, the strength of our study is the accuracy of PD diagnosis. The dataset has been used in Korea to establish a rare intractable disease (RID) enrollment program for reducing the economic burden of treating RIDs. PD has been registered as a RID since 2006, and, when registered, a special diagnostic code (V code) is assigned in addition to the ICD-10 code. The inclusion of PD (V124) in the RID registry is based on the following diagnostic criteria: (1) bradykinesia and at least one of the following symptoms: resting tremor, rigidity, and postural instability; (2) absence of exclusion criteria for PD; (3) three or more supportive prospective positive criteria for a definite PD diagnosis: unilateral onset, presence of resting tremor, progressive disorder, persistent asymmetry mainly affecting the side of onset, excellent response to levodopa, severe levodopa-induced chorea, levodopa response for over 5 years, and clinical course of over 10 years. The V code is only provided when specific comprehensive diagnostic criteria, as determined by a qualified clinician, are met; this has been proven to be particularly reliable information. Furthermore, we attempted to improve the diagnostic accuracy of dementia in PD by applying a 1-year rule in patient selection to exclude dementia with Lewy bodies or other mixed-type dementia. Our study, therefore, adds to the current state of knowledge concerning dementia treatment in PD.

## Conclusion

The present study suggests that in Korea, dementia occurs relatively early following the diagnosis of PD. In PD, age is an independent risk factor for dementia. The majority of PD + D patients are prescribed anti-dementia medication as monotherapy, for which the most commonly used was donepezil.

## Supplementary Information

**Additional file 1: Supplementary Table 1**. Incidence of dementia over time in Parkinson’s disease.

## Data Availability

Data are available from the Korea National Health Insurance Sharing service Institutional Data Access/Ethics Committee (https://nhiss.nhis.or.kr/bd/ay/bdaya001iv.do) for researchers who meet the criteria for the access to confidential data. Researchers can apply for the National Health Insurance data sharing service upon approval by the Institutional Review Board of their institution. After a review by the Korea National Health Insurance Sharing Service Institutional Data Access/Ethics Committee, the authors are required to pay a data access fee and confirm that other researchers will be able to access the data in the same manner as the authors.

## Abbreviations

PD: Parkinson’s disease
KNHIS-SC: Korean National Health Insurance Service-Senior Cohort
AD: Alzheimer’s disease
AChEI: Acetylcholinesterase inhibitor
ICD-10: International Classification of Diseases, 10th version
RID: Rare intractable disease
