# Supplementary material for: Patterns of dementia treatment in older adults with Parkinson’s disease using nationwide medical claims data
Source: BMC Geriatr. 2022 Apr 22;22:353. doi: 10.1186/s12877-022-03028-0 (PMC9026646; doi:10.1186/s12877-022-03028-0)
Supplement: Supplementary file 1 — Additional file 1: Supplementary Table 1. Incidence of dementia over time in Parkinson’s disease. [file 12877_2022_3028_MOESM1_ESM.pdf]

Supplementary table 1 Incidence of dementia over time in Parkinson's disease

		All (N=3879)							
		60's		70's		80's or more		total	
	Quarter	Cum. No.	(Cum. %)	Cum. No.	(Cum. %)	Cum. No.	(Cum. %)	Cum. No.	(Cum. %)
+1 year	1Q	31	(3.3)	152	(6.8)	75	(10.7)	258	(6.7)
	2Q	69	(7.4)	312	(13.9)	138	(19.7)	519	(13.4)
	3Q	103	(11.1)	454	(20.2)	186	(26.6)	743	(19.2)
	4Q	146	(15.7)	594	(26.4)	235	(33.6)	975	(25.1)
+2 year	1Q	188	(20.2)	707	(31.4)	286	(40.9)	1181	(30.4)
	2Q	227	(24.4)	832	(37.0)	324	(46.3)	1383	(35.7)
	3Q	257	(27.6)	941	(41.8)	369	(52.7)	1567	(40.4)
	4Q	294	(31.6)	1040	(46.2)	403	(57.6)	1737	(44.8)
+3 year	1Q	336	(36.1)	1134	(50.4)	425	(60.7)	1895	(48.9)
	2Q	377	(40.5)	1212	(53.9)	446	(63.7)	2035	(52.5)
	3Q	398	(42.8)	1308	(58.2)	473	(67.6)	2179	(56.2)
	4Q	428	(46.0)	1383	(61.5)	494	(70.6)	2305	(59.4)
+4 year	1Q	451	(48.5)	1467	(65.2)	516	(73.7)	2434	(62.7)
	2Q	472	(50.8)	1548	(68.8)	540	(77.1)	2560	(66.0)
	3Q	505	(54.3)	1615	(71.8)	559	(79.9)	2679	(69.1)
	4Q	532	(57.2)	1684	(74.9)	576	(82.3)	2792	(72.0)
+5 year	1Q	563	(60.5)	1733	(77.1)	590	(84.3)	2886	(74.4)
	2Q	592	(63.7)	1782	(79.2)	605	(86.4)	2979	(76.8)
	3Q	614	(66.0)	1833	(81.5)	611	(87.3)	3058	(78.8)
	4Q	639	(68.7)	1877	(83.5)	620	(88.6)	3136	(80.8)
+6 year	1Q	659	(70.9)	1918	(85.3)	631	(90.1)	3208	(82.7)
	2Q	683	(73.4)	1951	(86.7)	639	(91.3)	3273	(84.4)
	3Q	706	(75.9)	1982	(88.1)	646	(92.3)	3334	(85.9)
	4Q	724	(77.8)	2007	(89.2)	657	(93.9)	3388	(87.3)
+7 year	1Q	744	(80.0)	2026	(90.1)	664	(94.9)	3434	(88.5)
	2Q	762	(81.9)	2047	(91.0)	668	(95.4)	3477	(89.6)
	3Q	774	(83.2)	2075	(92.3)	671	(95.9)	3520	(90.7)
	4Q	786	(84.5)	2096	(93.2)	677	(96.7)	3559	(91.8)
+8 year	1Q	805	(86.6)	2112	(93.9)	679	(97.0)	3596	(92.7)
	2Q	817	(87.8)	2131	(94.8)	682	(97.4)	3630	(93.6)
	3Q	830	(89.2)	2148	(95.5)	683	(97.6)	3661	(94.4)
	4Q	846	(91.0)	2166	(96.3)	688	(98.3)	3700	(95.4)
+9 year	1Q	857	(92.2)	2180	(96.9)	688	(98.3)	3725	(96.0)
	2Q	869	(93.4)	2192	(97.5)	692	(98.9)	3753	(96.8)
	3Q	882	(94.8)	2207	(98.1)	695	(99.3)	3784	(97.6)
	4Q	891	(95.8)	2214	(98.4)	698	(99.7)	3803	(98.0)
+10 year	1Q	896	(96.3)	2218	(98.6)	698	(99.7)	3812	(98.3)
	2Q	907	(97.5)	2223	(98.8)	698	(99.7)	3828	(98.7)
	3Q	912	(98.1)	2229	(99.1)	699	(99.9)	3840	(99.0)
	4Q	916	(98.5)	2237	(99.5)	699	(99.9)	3852	(99.3)
+ 11 year	1Q~	930	(100.0)	2249	(100.0)	700	(100.0)	3879	(100.0)

Abbreviations: *Cum.* Cumulative, *No.* number
